# Rapid Integration of Zika Virus Prevention Within Sexual and Reproductive Health Services and Beyond: Programmatic Lessons From Latin America and the Caribbean

**DOI:** 10.9745/GHSP-D-18-00356

**Published:** 2019-03-22

**Authors:** Skye Beare, Emma Simpson, Kate Gray, Denitza Andjelic

**Affiliations:** a International Planned Parenthood Federation/Western Hemisphere Region, New York, NY, USA.; b International Planned Parenthood Federation, London, UK.

## Abstract

The emergence of the Zika virus (ZIKV) in Latin America and the Caribbean during the 2015–2016 outbreak has required local health systems to adapt, suddenly and continuously. This field action report explores the outbreak's sexual and reproductive health (SRH) complexities and the experience of the International Planned Parenthood Federation (IPPF) and 4 of its member associations as part of the United States Agency for International Development's rapid response in the region. It outlines approaches and actions that IPPF and its member associations undertook over 3 broad programmatic phases—developing ZIKV protocols and training personnel; delivering ZIKV-integrated services and information; and providing screening, care, and support for children and families affected by congenital Zika syndrome (CZS)—as the project worked to integrate ZIKV prevention, screening, and response within SRH service delivery models. It also describes the challenges and lessons learned in implementing a ZIKV response program in the region and recommendations from a service delivery perspective that can be useful in informing the responses to future rapid onset epidemics with SRH relevance. Challenges identified include adapting to a rapidly evolving evidence base during the early stages of the epidemic; traditional and restrictive regional social norms around gender, sex, and sexuality; the lack of focus on sexual transmission in national ZIKV public health messaging; and a lack of services, government support, and referral pathways for supporting children and families affected by CZS. Some of the key recommendations include finding ways to share rapidly evolving clinical updates conveniently and frequently, such as through digital technologies and platforms; partnering with multidisciplinary organizations, such as disability rights and services organizations, that can fill gaps in needed services; and leveraging the need for urgent action as a catalyst of change around more inclusive and gender-transformative social norms and services.

## Abstract (precis)

During the 2015–16 Zika virus outbreak, IPPF member association providers reached clients and affected populations faster by integrating critical information and services within existing sexual and reproductive health platforms. Challenges included: (1) communicating rapidly evolving evidence to providers; (2) overcoming restrictive social norms on gender and sexuality and a related lack of public messaging on preventing sexual transmission; and (3) addressing disability stigma and breaching service gaps to support children and caregivers affected by congenital Zika syndrome.

## BACKGROUND

The Zika virus (ZIKV) was first identified in Uganda in 1947 by scientists researching yellow fever in rhesus monkeys and subsequently isolated from humans in Uganda and Tanzania in 1952.^1^ Relatively small outbreaks occurred in Micronesia in 2007 and French Polynesia in 2014, but much remained unknown about the virus and its effects until more recently. It is primarily transmitted via *Aedes species* mosquitoes, including *Aedes aegypti* and *Aedes albopictus*, but can also be transmitted vertically from mother to embryo or fetus during pregnancy or at some point around the time of birth; sexually from partner to partner; and perhaps, though it seems rare and more evidence is needed, by blood transfusion and via health care setting or laboratory exposure.^2^ The virus has been detected in breast milk, vaginal fluids, urine, blood, and saliva and has been shown to remain the longest in semen, impacting the period of time it might be transferred to a sex partner.^3^ The Box provides more detail about the known transmission methods.

BOX **Zika Virus Transmission Methods**

**Mosquitoes**

Zika virus is principally transmitted to humans via the bite of an infected Aedes species mosquito, including *Aedes aegypti* and *Aedes albopictus*.

**Sexual Transmission and Bodily Fluids**

Zika virus has been detected in vaginal fluid, blood, semen, urine, saliva, and breast milk. No known cases of transmission via urine, saliva, or breast milk have been recorded. A person that has been infected with Zika can transmit the virus to a partner sexually. The virus remains in semen longer than in other bodily fluids.

**Vertical Transmission**

Mothers can pass Zika virus to the embryo or fetus during pregnancy or around the time of birth, though the exact mechanisms by which these types of transmission occur are still unknown.

**Blood, Blood Products, and Laboratory Exposure**

A limited number of cases of Zika virus transmission via blood transfusion and laboratory exposure have been identified. No known cases of transmission via organ transplantation have been recorded.

Sources: CDC (2019),^2^ Gregory (2017).^4^

Largely asymptomatic, the most devastating effects of ZIKV are now known to originate during pregnancy with the potential to severely affect embryonic and fetal development. These effects can include the development of congenital microcephaly and a range of neurological, motor, auditory, visual, and other delays and abnormalities now referred to collectively as congenital Zika syndrome (CZS),^5^ with far-reaching consequences for sufferers and caregivers alike. Guillain-Barré syndrome has also been strongly associated with Zika.^6^

A major ZIKV outbreak in Latin America and the Caribbean between 2015 and 2016, the first of its scale, prompted the World Health Organization (WHO) to formally declare a temporary “Public Health Emergency of International Concern” in the region. Now considered endemic to the region, as of January 2018 authorities had recorded 806,701 cumulative confirmed and suspected autochthonous ZIKV cases across 48 countries in the region, with 3,720 confirmed instances of CZS.^7^

In Latin America and the Caribbean, sexual and reproductive rights are severely curtailed, more than half of all pregnancies are unintended,^8^ and many lack even basic access to quality sexual and reproductive health (SRH) care. Given that ZIKV can be sexually transmitted and has the ability to negatively affect pregnancy outcomes, it is evident that the burden of the virus in this region rests primarily on women and girls of reproductive age. This required a coordinated response that included a specific focus on sexual and reproductive health and rights, with tailored interventions. The United States Agency for International Development (USAID) was one of several actors, including the Pan American Health Organization (PAHO), WHO, and the United Nations Population Fund (UNFPA), that acted quickly to mitigate the spread and impact of ZIKV, especially among women and girls of reproductive age.

The burden of Zika in Latin America and the Caribbean rests primarily on women and girls of reproductive age.

In June 2016, through the Support for International Family Planning Organizations 2 – Sustainable Networks Project (SIFPO2), International Planned Parenthood Federation (IPPF) member associations in 4 priority countries (the Dominican Republic, El Salvador, Guatemala, and Honduras) became implementing partners in USAID's ZIKV rapid response efforts. These organizations worked together in close coordination with several USAID cooperating agencies and partners supporting complementary efforts, including the Applying Science to Strengthen and Improve Systems (ASSIST) Project, the Maternal and Child Survival Program, the Knowledge for Health (K4Health) Project, the Health Communication Capacity Collaborative (HC3), and Population Services International (PSI). While the overall USAID response was broader, incorporating vector control and policy development among other initiatives, IPPF's involvement focused especially on improving access to and delivery of quality ZIKV-integrated SRH services, including voluntary family planning for women and girls of reproductive age.

The principal goal of the SIFPO2 Zika Prevention Project was to integrate ZIKV prevention information within existing SRH services and outreach as quickly as possible (a rapid integration approach) to support and strengthen health systems in the 4 priority countries in order to minimize negative pregnancy outcomes. It aimed to do so by improving access to ZIKV-integrated SRH and child health services, including voluntary family planning services and counseling, and by improving providers' capacity to deliver ZIKV-integrated services and information.

In 2016, IPPF and its local member associations led a Zika prevention project in 4 priority Latin America and Caribbean countries.

IPPF and its regional entity, the International Planned Parenthood Federation/Western Hemisphere Region (IPPF/WHR), played key organizational, facilitating, and convening roles in the implementation of the project, providing continual technical and project management assistance to the member associations.

IPPF's member associations in each of the 4 countries have well-established networks of trained service providers operating from a range of service delivery points, from static clinics to mobile outreach services, and in some cases in partnership with government outlets. Member associations routinely work collaboratively with private- and public-sector health care providers and counterparts to refer clients to specialized services, depending on local needs and capacities. By effectively deploying and expanding these networks, the member associations—trusted local providers of a wide range of health services—were able to reach large numbers of at-risk women and men. Efforts focused particularly on (1) pregnant women who wished to avoid sexual transmission from a partner and (2) women who wished to delay or limit pregnancies, including women with an unmet need for family planning.

**FIGURE 1:**
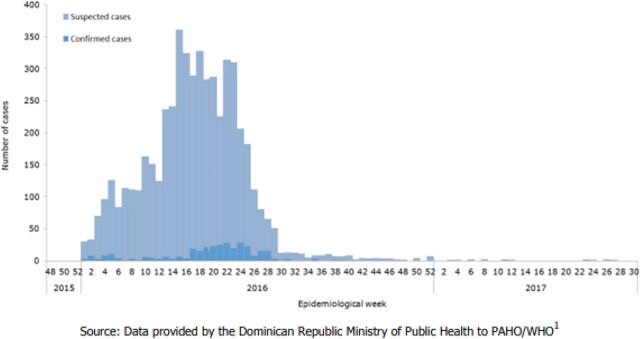
Suspected and Confirmed Zika Virus Cases, Dominican Republic, Epidemiological Week 48 of 2015 to Epidemiological Week 30 of 2017 Source: PAHO and WHO (2017).^9^

**FIGURE 2:**
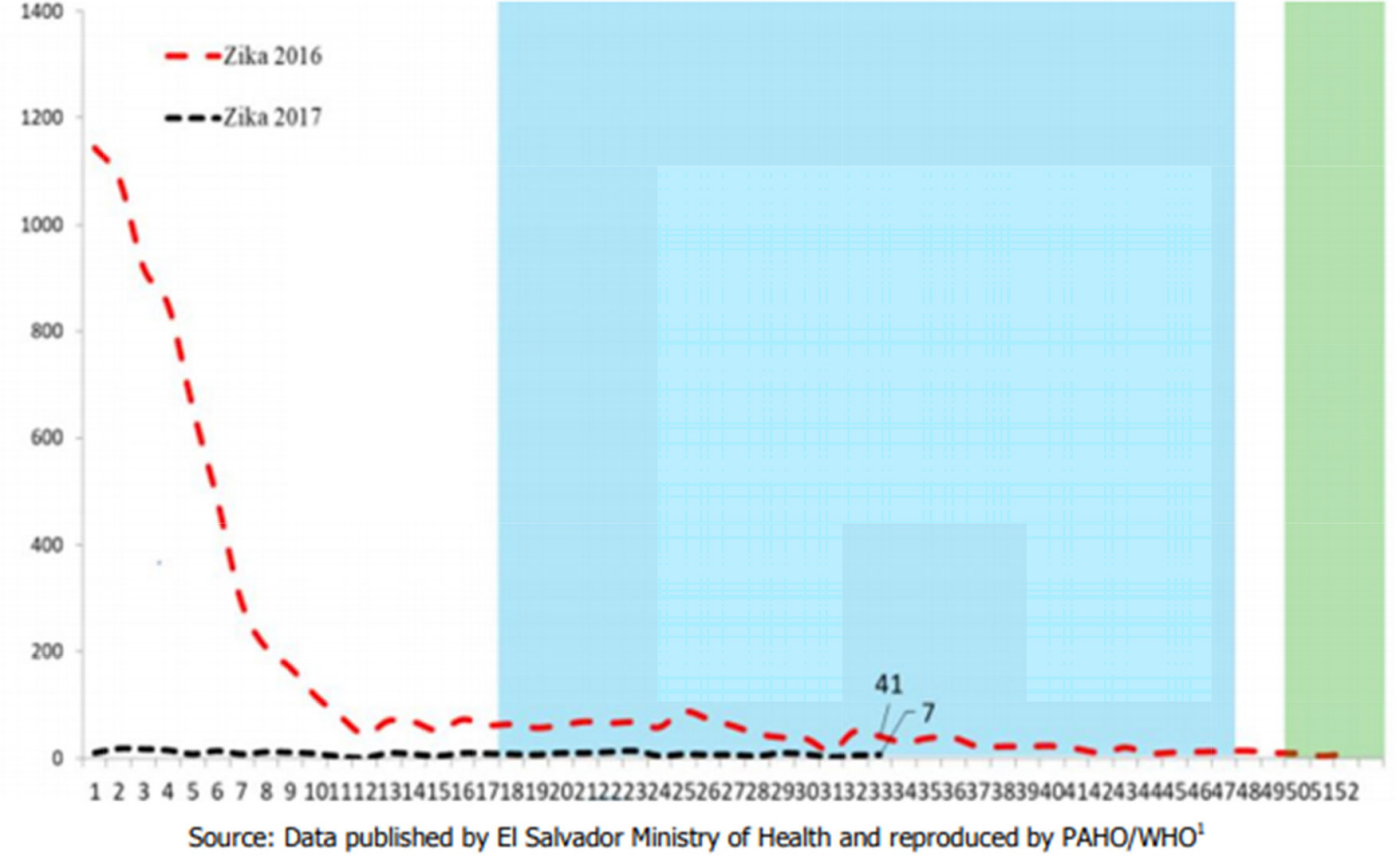
Suspected and Confirmed Zika Cases, El Salvador, Epidemiological Week 1 of 2016 to Epidemiological Week 33 of 2017 Source: PAHO and WHO (2017).^10^

**FIGURE 3:**
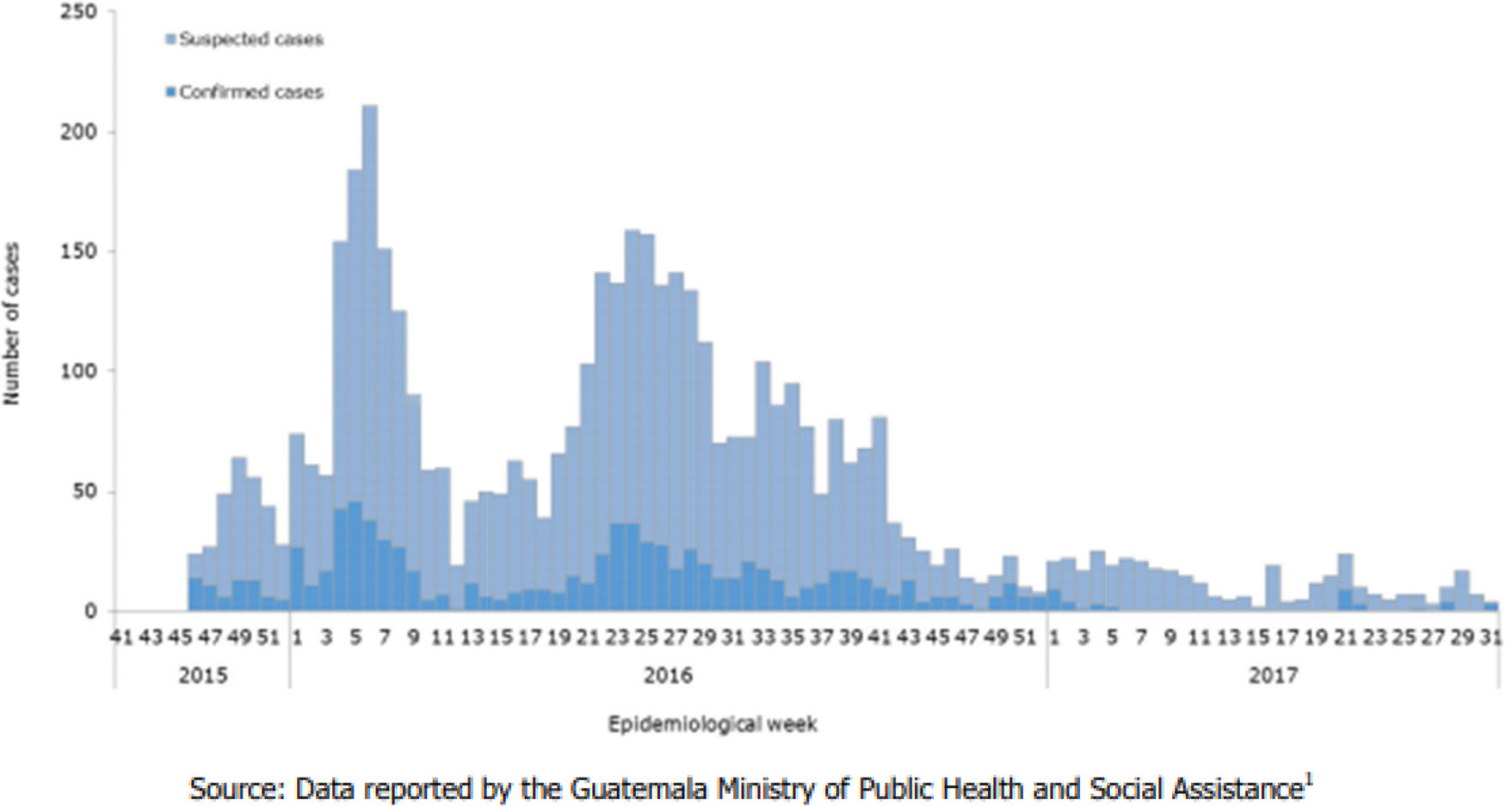
Suspected and Confirmed Zika Cases, Guatemala, Epidemiological Week 41 of 2015 to Epidemiological Week 31 of 2017 Source: PAHO and WHO (2017).^11^

**FIGURE 4:**
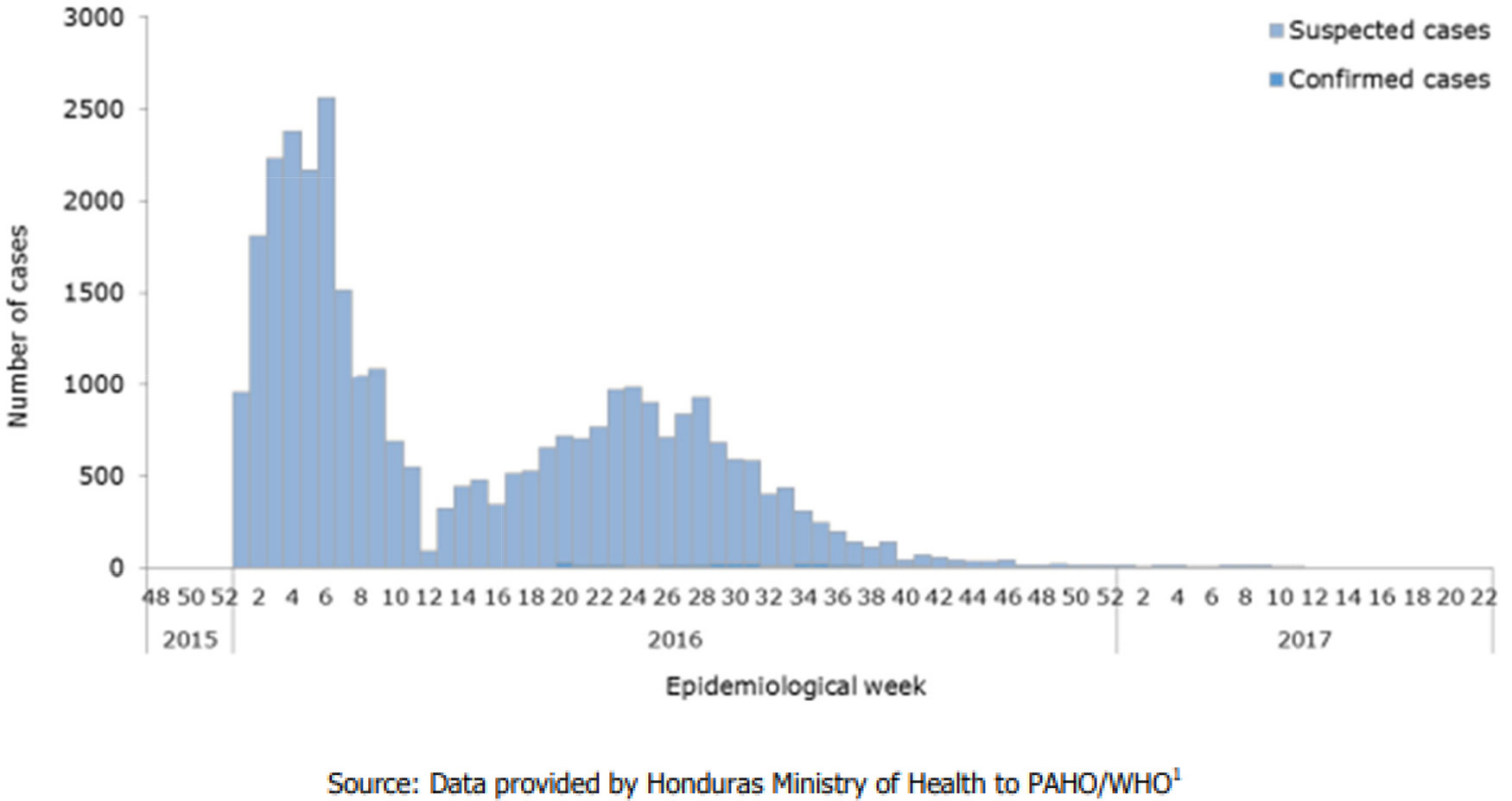
Suspected and Confirmed Zika Virus Cases, Honduras, Epidemiological Week 48 of 2015 to Epidemiological Week 22 of 2017 Source: PAHO and WHO (2017).^12^

A summary of the 4 member associations' general reach and services is provided in the Table, and a visual representation of the ZIKV epidemic curve between 2015/2016 and 2017 from PAHO/WHO epidemiological reports is available in Figure 1 for the Dominican Republic, Figure 2 for El Salvador, Figure 3 for Guatemala, and Figure 4 for Honduras.

**TABLE.** Snapshot of IPPF Member Associations' Institutional Size and Scope in Each Country, 2017

Country	Member Association Name	Service Delivery Points	Estimated No. of Clients, 2017	No. of FP Services, 2017	No. of SRH Services, 2017	No. of FP Commodity Units Provided, 2017
Dominican Republic	Asociación Dominicana Pro-Bienestar de la Familia (Profamilia)	2 associated clinics 28 CBD outlets 92 commercial marketing outlets 5 government outlets 2 mobile clinics 6 other agencies 657 private physicians 7 static clinics	163,940	73,568	637,023	2,218,855
El Salvador	Asociación Demográfica Salvadoreña (ADS)/Pro-Familia	999 CBD outlets 506 commercial marketing outlets 8 mobile clinics 17 other agencies 71 private physicians 13 static clinics	251,660	666,807	1,599,958	2,017,038
Guatemala	Asociación Pro-Bienestar de la Familia de Guatemala (APROFAM)	2,000 CBD outlets 5 mobile clinics 26 static clinics	327,250	513,400	1,398,973	724,418
Honduras	Asociación Hondureña de Planificación de Familia (ASHONPLAFA)	95 associated clinics 1,701 CBD outlets 1,014 commercial marketing outlets 1 government outlet 19 mobile clinics 32 static clinics	775,880	211,129	2,224,952	2,175,489

Abbreviations: CBD, community-based distribution; FP, family planning; IPPF, International Planned Parenthood Federation; SRH, sexual and reproductive health.

## PROJECT DESIGN AND IMPLEMENTATION

**FIGURE 5:**
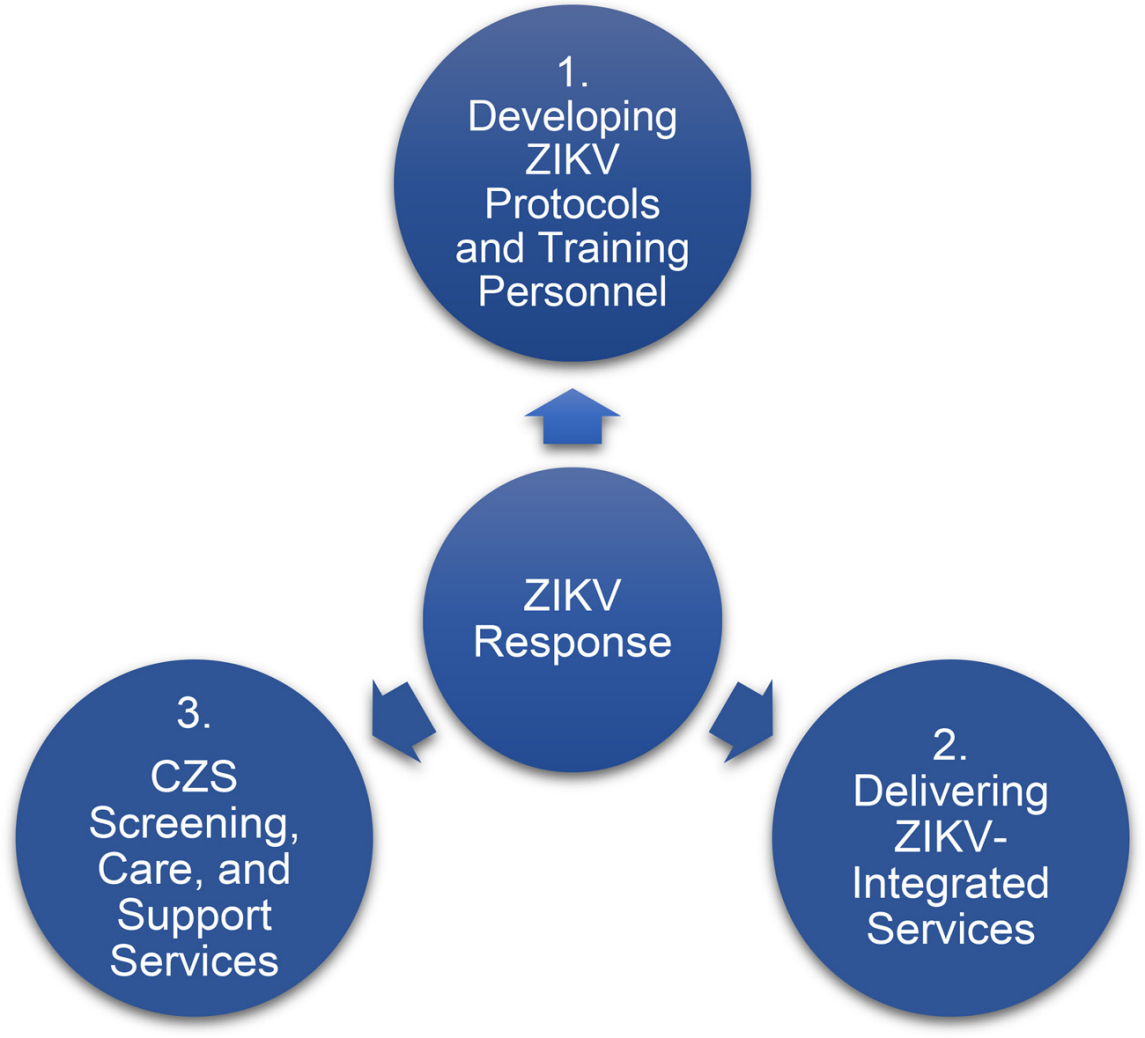
Programmatic Components of the IPPF SIFPO2 Zika Prevention Project in Latin America and the Caribbean Abbreviations: CZS, congential Zika syndrome; IPPF, International Planned Parenthood Federation; SIFPO2, Support for International Family Planning Organizations 2 – Sustainable Networks Project; ZIKV, Zika virus.

The project consisted of 3 broad programmatic phases as indicated in Figure 5, beginning with the initial and adaptive development of ZIKV protocols and training of personnel and continuing with the direct implementation and delivery of ZIKV-integrated SRH services. Initially, the project focused on integrating the newly adapted ZIKV protocols during family planning visits, antenatal and postnatal care consultations, and community education efforts. Later, as the true scope of ZIKV implications became better understood, information was also integrated into all SRH and gynecological services in order to reach as many women and girls as possible across the continuum of care. Each member association tailored its response and specific services to its respective country context, institutional capacity, and organizational strengths. Finally, the project incorporated an expanded focus on the development of screening, care, and support services for children and families affected by CZS during postnatal screenings and early well-child visits, where available. The approaches, challenges, and lessons learned during each of the 3 programmatic phases are discussed below in more detail.

### Capacity Building: Developing ZIKV Protocols and Training Personnel

A critical training phase at the outset of the project ensured that IPPF member association clinical staff and community health worker (CHWs) had sufficient skills and knowledge to deliver accurate, high-quality ZIKV-related services and information to at-risk clients. This required developing a standardized set of ZIKV integration and response protocols and training curricula to guide clinical teams and CHWs. These were then updated on a rolling basis as scientific understanding of the virus' pathology improved. Under the leadership of ASSIST, IPPF member associations worked closely with Ministry of Health officials and other implementing partners to support development and subsequent adaptations of these essential tools. ASSIST developed a package of materials including job aids, protocols, and service algorithms to guide preconception, antenatal, and family planning counseling in the context of ZIKV. PSI developed a regional communication campaign to promote the most effective ways of preventing ZIKV, and IPPF member associations also developed context-specific information, education, and communication (IEC) materials tailored to reach local communities.

Member associations launched trainings based on the new protocols and guidelines, customized for each cadre of health worker involved in the response, including direct service providers (obstetricians/gynecologists, general practitioners, counselors, psychologists), educators, and community family planning distributors and other CHWs. The content of the training included virus pathology, symptoms and prevention, voluntary and locally available family planning methods, and the newly developed protocols. Several subsequent rounds of training were provided as protocols were revised and understanding about the epidemic and its consequences evolved. By the end of the project's total 23-month period of performance, 5,298 providers of all types had been trained at least once, with 1,786 reporting having received at least one round of updated or refresher training beyond the initial session.

#### Challenges

**A rapidly evolving evidence base and the need for ongoing training.** The rapidly expanding ZIKV evidence base, and particularly the emerging consensus regarding the importance of early detection of CZS, required IPPF member associations and other partners to remain flexible and responsive, quickly adapting information and messaging for IEC materials, revising protocols, and conducting multiple rounds of training for service providers. This was a significant challenge for clinical directors who had to balance a range of competing demands on providers' time in scheduling trainings. To keep providers informed of the evolving evidence and best practice while reducing the burden of participating in multiple in-person trainings, the IPPF member association in the Dominican Republic, Profamilia, developed a community of practice website that providers could access individually and on their own time, helping to ease the burden.

Because the evidence base on Zika was rapidly evolving, IPPF and its partners had to quickly adapt messaging, revise protocols, and train service providers multiple times.

#### Lessons and Recommendations

**Balancing competing priorities.** Organizations responding to public health emergencies like the ZIKV outbreak must remain agile and able to respond quickly yet accurately as new evidence emerges. Conducting research, performing assessments, coordinating partners, and developing standardized care protocols and guidelines as part of a comprehensive approach require time, and yet the need to reach communities with critical information and services is urgent. Achieving this balance of speed and accuracy can be difficult, so attempts to anticipate and mitigate bottlenecks in advance are helpful. Thinking creatively of ways to alleviate the impact of the additional continuous training demands on providers wherever resources and local context allow also helps to mitigate competing demands. Utilizing digital trainings and platforms to share clinical updates and reinforce in-person trainings also allows for repeated exposure to key themes and bolsters learning. Similar programs should anticipate the need for ongoing facilitation of updates to protocols and training programs from the outset, which will improve service quality and strengthen the long-term impact of the initial response.

### Prevention: Delivering ZIKV-Integrated Services and Information

The second phase of implementation involved the delivery of ZIKV-integrated services and comprehensive information to the most vulnerable, including women and girls in areas highly affected by ZIKV, those most economically disadvantaged, and pregnant women and their partners. The focus of this phase of work centered on the prevention of unintended pregnancies by improving access to voluntary family planning information and services and on the prevention of sexual transmission of ZIKV for both pregnant and non-pregnant individuals. To address financial barriers to access, relevant services were subsidized in part or in full, depending on client need in target areas and individual member association approaches. A total of 216,091 clients at project sites accepted a voluntary family planning method, resulting in the provision of an estimated 185,444 couple-years of protection (CYP) and the aversion of an estimated 88,195 unintended pregnancies, as estimated using the Marie Stopes International Impact 2 calculator.^13^

Outside of clinical settings, educators and community family planning distributors focused on increasing awareness about ZIKV prevention and voluntary family planning among vulnerable and remote communities. Trained CHWs provided short-acting methods of contraception including oral contraceptives and condoms and referred clients to clinics to receive long-acting and permanent methods as requested per clients. Community distributers and educators also provided up-to-date information about ZIKV, how to prevent sexual transmission of the virus, and locally available voluntary family planning methods. In total, an estimated 674,287 individuals in project sites received ZIKV-integrated SRH information. IEC sessions were adapted according to target groups and settings:

- In Honduras, the Asociación Hondureña de Planificación de Familia (ASHONPLAFA) worked in partnership with bus companies, palm oil processing plants, and fast food outlets to educate workers on the job. Community educators delivered health information sessions in the workplace, sometimes *while* people worked or during extended lunch breaks, ensuring that essential information on ZIKV and locally available family planning options was shared as widely as possible.
- In El Salvador, the Asociación Demografica Salvadoreña (ADS)/Pro-Familia leveraged its regular contributions to a local radio show about relationships and SRH called *Confiésame Love* (translated roughly as “Love Confessions”) to deliver ZIKV messaging to listeners. They also distributed small cosmetic bags filled with IEC materials and condoms to pregnant women, a discreet effort tailored to the local context that worked to promote the use of barrier methods during pregnancy.
- In Guatemala, the Asociación Pro-Bienestar de la Familia (APROFAM) made ZIKV a central topic at discussion clubs for pregnant mothers (*Club de la Amiga de la Embarazada*) to assist women in understanding the risks of ZIKV during pregnancy and how to prevent transmission or access support as necessary.

**Figure:**
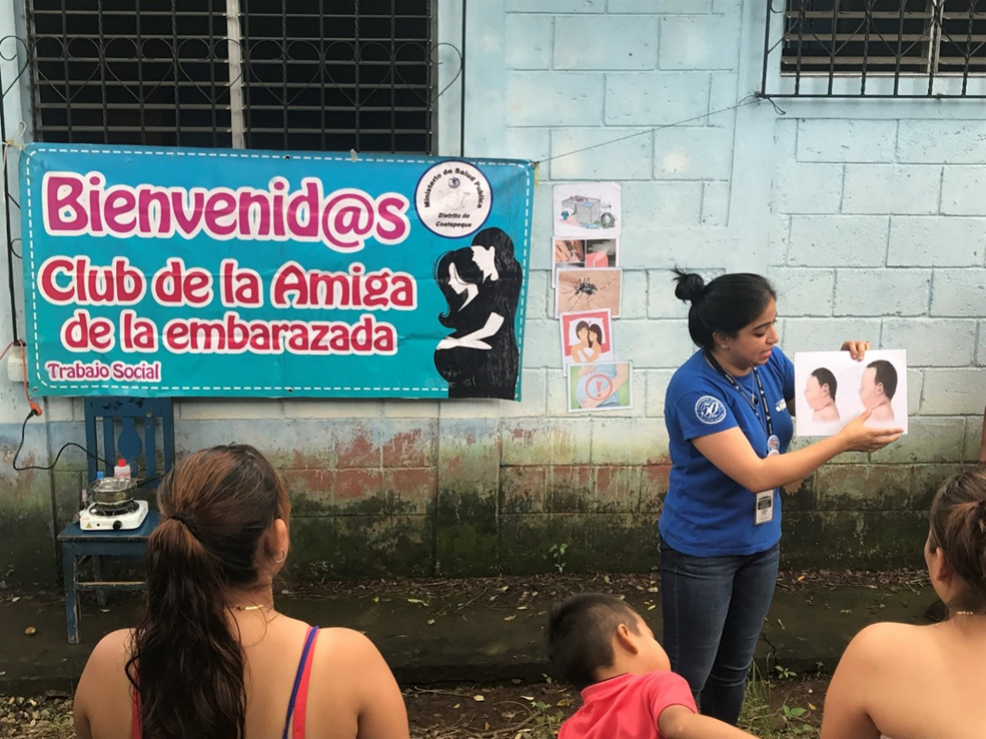
An APROFAM community health worker in Guatemala teaches members of a community-based discussion club for pregnant women about the risks of Zika and corresponding prevention strategies. © 2017 Skye Beare/IPPFWHR

- In the Dominican Republic, mobile health units brought ZIKV-integrated services and language appropriate information sessions to remote, rural *bateye* communities of vulnerable Haitian immigrants that otherwise traditionally experience geographic access barriers to care and information.
- Because men and boys are affected by, involved in, and to varying degrees may control decision making and behaviors that affect SRH and contraceptive use,^14^^,^^15^ their inclusion was also a critical component of the ZIKV response. In Honduras, ASHONPLAFA brought facts and judgment-free information on ZIKV-integrated SRH to an estimated 81,000 men in total, 58,000 of whom were reached via traditionally male-dominated spaces like military outposts, sports clubs, Scouts meetings, and tobacco-processing plants.

#### Challenges

**Overcoming restrictive social norms.** Traditional religious, gender, and cultural norms and stigma in Latin America and the Caribbean often reinforce negative attitudes toward sex and sexuality, discourage contraceptive use, and place significant value on the role of women—including even young women—as mothers. In this context, the delivery of culturally sensitive but also gender transformative, sex positive, and medically accurate information about SRH is particularly challenging. The ZIKV epidemic created new opportunities to initiate dialogue about the value of family planning; however, in other ways it created new communication challenges. For example, messaging about the need to use condoms *during pregnancy* to prevent sexual transmission was met with some confusion and resistance from both men and women.

**Lack of comprehensive public health messaging on sexual transmission.** Because of the restrictive SRH and rights landscapes in project countries and in the region, public-sector responses focused primarily on vector control in their effort to halt the virus' spread, to the near total exclusion of investment in prevention of sexual transmission. National responses to the crisis like the one in El Salvador,^16^ for example, have been underfunded and incomplete in merely instructing women to avoid or delay pregnancy without addressing the existing unmet need for family planning. Lack of public awareness surrounding the risks and ongoing transmission of ZIKV and the importance of using barrier methods to prevent sexual transmission persists.

Because of the restrictive sexual and reproductive health landscape in the region, public-sector responses to Zika focused primarily on vector control with little attention on preventing sexual transmission of the virus.

Between July and August 2017, ASHONPLAFA conducted an integrated ZIKV virus and family planning Knowledge, Attitudes and Practices (KAP) study among a sample of individuals accessing health services across a range of its static clinics to better understand this persistent lack of awareness. With a sample of 620 people, the KAP survey reported that while 94% had prior knowledge and awareness of ZIKV transmission via mosquito bite, only 43% had knowledge of sexual transmission, with a mere 14% reporting having taken any form of precaution to prevent transmission. Further, only 48% reported awareness of the link between ZIKV and microcephaly. In part, this can be attributed to a lack of comprehensive messaging about sexual transmission of ZIKV and the lack of prioritization of family planning in the public sector as part of a comprehensive response. Public awareness campaigns commonly excluded information about sexual transmission and its prevention, and cultural and political sensitivities appear to have prevented a strong public focus on this aspect of the epidemic. Consequentially, community providers and clinicians who rely on the public sector to inform key populations of health threats struggled to change perceptions.

#### Lessons and Recommendations

**Transformative gender approach.** Every public health or development challenge, whether local, regional, or global in scope, in the context of an emergency or otherwise, also contains within it an inherent opportunity to challenge gendered societal inequalities. Adopting a gender transformative approach that actively promotes gender equality can help interventions to counter restrictive social norms and attitudes that inform and ultimately compromise the scientific integrity, accuracy, reach, and effectiveness of public health responses.

**Improving access by removing barriers.** Reducing financial barriers to access for clients in target communities was essential to increasing uptake of voluntary family planning services. While information on ZIKV has been integrated within target services, the completion of the project means each member association's ability to sustain and expand continuing provider trainings, ongoing community education activities, and—critically—subsidies for poor, young, vulnerable, and otherwise at-risk populations is negatively affected. Funding subsidized family planning services where they are not already available should be a priority for governments and donors in the region.

IPPF and its member associations champion a client-centered approach to delivering SRH services and recognize that negative client experiences can also create barriers to access. After surveying a total of 812 individual clients in the Dominican Republic, Guatemala, and Honduras using an adapted client satisfaction survey tool called the Net Promoter Score, member associations were able to use the results to directly improve service provision. For example, in the Dominican Republic, clients identified long wait times as an area of concern and potential barrier to access, so Profamilia revised its patient flow procedures and reduced the wait times. In Guatemala and Honduras, clinic directors identified services in which fewer clients reported receiving ZIKV-related information and then facilitated additional provider trainings and support to ensure more clients received this critical information. Insisting that clients are always at the center of the intervention and their feedback is not just heard but acted upon improves experiences, encouraging increased health-seeking behaviors.

### Response: CZS Screening, Care, and Support Services

A third phase of work focused on addressing the needs of children and families affected by ZIKV and CZS. Significant gaps exist in public health systems across Latin America and the Caribbean for services for people living with disabilities, CZS included. Research has shown that response to treatment in infants affected by CZS is dependent on not only the severity of their complications but also how early they access treatment and essential therapies.^17^ It was therefore critical that each member association also integrate ZIKV and CZS to the extent possible within its available postnatal and child wellness services.

Working closely with ASSIST, project teams in El Salvador and Honduras incorporated CZS-specific information into service provider guidelines for postnatal and early well-child health check-ups, with the aim of detecting delays and deficits that are not always apparent at birth and might only appear later, as children fail to meet standard developmental markers. These screenings provided an opportunity to detect possible cases and to encourage positive health-seeking behavior during and after the outbreak. In Guatemala, the team hired 2 psychologists to work with infants and address the psychosocial needs of mothers and families affected by CZS, conducting routine home visits to help reach remote, rural households who otherwise struggled to access care. These personnel provided essential assessment and referral capacity to existing though limited therapeutic services through regular monitoring and early well-child health follow-ups.

#### Challenges

**Service gaps and disability stigma.** With critical gaps in public health systems across the region and limited existing referral pathways, the IPPF member associations struggled to link families to a full range of essential resources and services such as therapy and rehabilitation centers. Disability stigma also had an impact on parents and families bringing their infants for treatment and support services, particularly those with microcephaly and other visible CZS outcomes. Some member associations reported cases of parents who would obscure their baby's heads with hats during checkups in an effort to avoid detection and diagnosis, underscoring the need to bring psychosocial support to caregivers and destigmatizing IEC efforts to wider communities.

Stigma around disability prevented some parents of infants with microcephaly or other visible effects of Zika from bringing their infants for treatment and support services.

#### Lessons and Recommendations

**Adopting broad, multidisciplinary approaches to reach at-risk populations.** One way to simultaneously fill such gaps, tackle stigma, and reach especially vulnerable populations is to expand this work in a multidisciplinary manner across mixed health systems by partnering with organizations that can provide necessary but missing expertise, access, or coverage. For example, in Guatemala, APROFAM forged partnerships early in the project with local disability rights and services organizations to ensure incorporation of their specialist expertise and input throughout the design and delivery of the response. By partnering with these organizations and taking a holistic approach to the entire life cycle of SRH care that included early child wellness, the member association was able to reach more women and families with infants affected by CZS and other disabilities. Acknowledging systemic gaps in capacity early and engaging in partnerships with disability-focused organizations and other specialists will over time and with continued advocacy help to foster the growth of necessary support mechanisms. This will also help to center the voices and experiences of groups that traditionally experience high levels of stigma or discrimination as stakeholders and active participants within a given intervention, and not just as external beneficiaries.

Similarly, in Honduras, ASHONPLAFA worked to establish ties to local Afro-Caribbean Garifuna populations, bringing culturally sensitive mobile outreach to distant communities, and translated ZIKV-integrated SRH IEC messages to radio stations in the local language. In a humanitarian context, working collaboratively in multidisciplinary partnerships can help leverage limited resources and ensure the broadest, fastest, most inclusive possible reach of programmatic interventions.

More generally, incorporating a broad health systems strengthening approach to ensure all populations affected by a given public health challenge are reached with appropriate interventions may also be required. While the early stages of the project (Phases 1 and 2) focused primarily on integrating ZIKV prevention and support within existing SRH services, Phase 3 of the project saw IPPF member associations expand support within the primary health care setting to reach families and children at various points across the health system. The specific nature of the ZIKV and its consequences and relevance to SRH care as well as child health care necessarily required adaptations across health systems. NGOs and donors alike have a responsibility to consider and promote these cross-cutting adaptations wherever possible.

## CONCLUSION

The ZIKV outbreak in Latin America and the Caribbean posed a new public health threat to the region and globally. The scale of the epidemic, limited disease knowledge at the outset, and the constantly evolving context presented challenges that had to be overcome with adaptive and innovative approaches. It also exposed existing gaps and weaknesses in local and national health systems.

The international community, governments, and public health actors at all levels must recognize the ongoing threat that ZIKV continues to pose globally. Globalized travel, environmental factors including climate change that increases the range of disease vectors, increasingly variable rainy seasons and flooding that create exponential increases in levels of standing water and mosquito hatches, and the vast quantity of information that is still unknown about the virus' pathology mean future outbreaks are highly possible. It is worth noting that the same climate change-caused flooding that is projected to result in 250,000 additional deaths per year between 2030 and 2050 from malaria and other causes^18^ could also result in both more frequent and more widespread ZIKV outbreaks. Reponses to such emergencies should be designed in a manner that affirms the sexual and reproductive health and rights of women and girls, acknowledging the specific contexts in which such outbreaks occur including existing barriers to health, who is most at risk, and who bears the burden of preventing negative outcomes. Working in partnership across multidisciplinary public, private, and nonprofit sector divisions will facilitate expanded intervention coverage and ensure expanded inclusivity, particularly of vulnerable populations.

Finally, all humanitarian emergencies that affect people's ability to enjoy the full range of sexual and reproductive health and rights should be addressed as such, whether through an independent effort or as part of a more comprehensive approach. A successful response to another, future ZIKV outbreak or similar public health threat—or any humanitarian or emergency situation—will recognize this and leverage the need for urgent action as a catalyst of change around more inclusive social norms and services.

## Notes

**En español**

**Integración rápida de la prevención del virus del Zika en los servicios de salud sexual y reproductiva y más allá de estos: lecciones programáticas desde América Latina y El Caribe**

Durante el brote del virus del Zika, en 2015-2016, los proveedores de las Asociaciones Miembro llegaron a clientes y a poblaciones afectadas con mayor rapidez al integrar información y servicios fundamentales dentro de las plataformas existentes de salud sexual y reproductiva. Algunos de los desafíos fueron: (1) comunicar rápidamente la evidencia cambiante a los proveedores; (2) superar las normas sociales restrictivas sobre el género y la sexualidad, y la relativa falta de mensajes públicos para prevenir la transmisión sexual; y (3) abordar el estigma por discapacidad y cerrar las brechas de servicios para apoyar a los niños y niñas y a personas a cargo de su cuidado, afectadas por el síndrome congénito del Zika.

**RESUMEN**

La emergencia del virus del Zika (VZIK) en América Latina y el Caribe durante el brote de 2015-2016 requirió que los sistemas locales de salud se adaptaran, de manera abrupta y continua. Este informe de acción de campo explora las complejidades del brote en materia de salud sexual y reproductiva (SSR) y la experiencia de la Federación Internacional de Planificación Familiar (IPPF) y 4 de sus Asociaciones Miembro, que formaron parte de respuesta rápida de la Agencia de los Estados Unidos para el Desarrollo Internacional en la región. Describe los enfoques y las acciones que siguieron la IPPF y sus Asociaciones Miembro durante tres fases programáticas amplias –elaborar protocolos sobre el VZIK y capacitar al personal; prestar servicios e información integrados al VZIK; y proveer servicios de detección, atención y apoyo a los niños y las familias afectadas por el síndrome congénito del Zika (SCZ) –puesto que el proyecto trabajó para integrar la prevención, la detección y la respuesta al VZIK dentro de los modelos de prestación de servicios de SSR. También describe los desafíos y las lecciones aprendidas en la implementación de un programa de respuesta al VZIK en la región y recomendaciones, desde una perspectiva de prestación de servicios, que pueden ser útiles para nutrir las respuestas ante epidemias de inicio rápido con relevancia para la SSR en la región. Los retos identificados abarcan una base de evidencias rápidamente cambiante durante las primeras etapas de la epidemia; las normas sociales tradicionales y restrictivas sobre género, sexo y sexualidad en la región; la falta de énfasis en la transmisión sexual del VZIK en los mensajes de salud pública; y la falta de servicios, apoyo gubernamental y rutas de derivaciones (referencias) para apoyar a los niños y a las familias afectadas por SCZ. Algunas de las recomendaciones esenciales incluyen encontrar alternativas para compartir de manera rápida y frecuente actualizaciones clínicas que están en constante cambio –por ejemplo, a través de tecnologías y plataformas digitales-; generar alianzas con organizaciones multidisciplinarias que puedan llenar los vacíos de servicios requeridos, como por ejemplo, las organizaciones de derechos y servicios para las personas con discapacidad; y aprovechar la necesidad de acciones urgentes que catalicen el cambio en materia de normas sociales y servicios más incluyentes y transformadores de género.

**Peer Reviewed**

**Funding:** The SIFPO2 Zika Prevention Project was funded by the United States Agency for International Development as part of the wider Support for International Family Planning Organizations 2 – Sustainable Networks Project (SIFPO2) Project.
